# Rapid determination of malondialdehyde in serum samples using a porphyrin-functionalized magnetic graphene oxide electrochemical sensor

**DOI:** 10.1007/s00216-023-04594-x

**Published:** 2023-02-20

**Authors:** Jihène Ben Attig, Latifa Latrous, Ismael Galvan, Mohammed Zougagh, Ángel Ríos

**Affiliations:** 1 Department of Analytical Chemistry and Food Technology, Faculty of Chemical Sciences and Technologies, University of Castilla-La Mancha, Campus Universitario, 13071 Ciudad Real, Spain; 2 Regional Institute for Applied Scientific Research, IRICA, Camilo José Cela Avenue, E-13005 Ciudad Real, Spain; 3 Laboratoire de Chimie Analytique Et Electrochimie, Department of Chemistry, Faculty of Sciences of Tunis, University of Tunis El Manar, University Campus of El Manar II, 2092 Tunis, Tunisia; 4 Laboratoire de Chimie Minérale Appliquée, Department of Chemistry, Faculty of Sciences of Tunis, University of Tunis El Manar, University Campus of El Manar II, 2092 Tunis, Tunisia; 5 Department of Evolutionary Ecology, National Museum of Natural Sciences, CSIC, 28006 Madrid, Spain; 6 Department of Analytical Chemistry and Food Technology, Faculty of Pharmacy, University of Castilla-La Mancha, 02071 Albacete, Spain

**Keywords:** Porphyrin-functionalized magnetic graphene oxide, Screen-printed carbon electrode, Electrochemical sensor, Malondialdehyde, Serum samples

## Abstract

An electrochemical sensor based on a screen-printed carbon electrode (SPCE) modified with porphyrin-functionalized magnetic graphene oxide (TCPP-MGO) was developed for the sensitive and selective determination of malondialdehyde (MDA), an important biomarker of oxidative damage, in serum samples. The coupling of TCPP with MGO allows the exploitation of the magnetic properties of the material for separation, preconcentration, and manipulation of analyte, which is selectively captured onto the TCPP-MGO surface. The electron-transfer capability in the SPCE was improved through derivatization of MDA with diaminonaphthalene (DAN) (MDA-DAN). TCPP-MGO-SPCEs have been employed to monitor the differential pulse voltammetry (DVP) levels of the whole material, which is related to the amount of the captured analyte. Under optimum conditions, the nanocomposite-based sensing system has proved to be suitable for the monitoring of MDA, presenting a wide linear range (0.01–100 µM) with a correlation coefficient of 0.9996. The practical limit of quantification (P-LOQ) of the analyte was 0.010 µM, and the relative standard deviation (RSD) was 6.87% for 30 µM MDA concentration. Finally, the developed electrochemical sensor has demonstrated to be adequate for bioanalytical applications, presenting an excellent analytical performance for the routine monitoring of MDA in serum samples.

**Graphical abstract:**

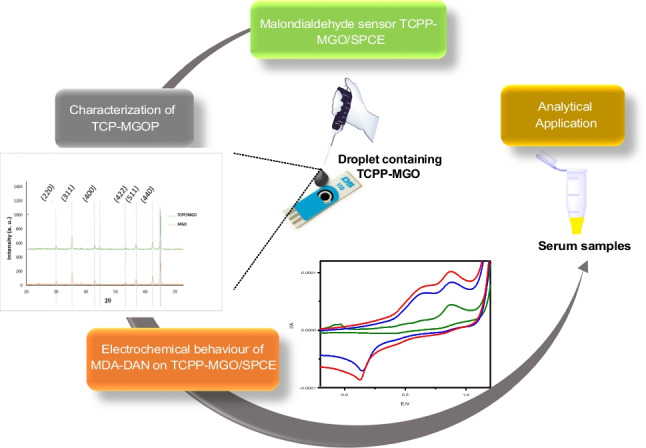

## Introduction

Lipid peroxidation is one of the most relevant consequences of free radicals, particularly reactive oxygen species (ROS), for biological systems. This process occurs when free radicals attack the constituent lipids of cell membranes, especially polyunsaturated fatty acids (PUFAs) [1]. Lipid peroxidation can produce changes in the permeability and fluidity of the membrane lipid bilayer and affect the cell integrity [2]. Malondialdehyde (MDA) is an end product of lipid peroxidation and, as such, an important biomarker of oxidative damage of PUFAs [3]. MDA also induces DNA damage. As a consequence, MDA represents an indirect indicator of different diseases such as cancers [4, 5], diabetes [6], cardiovascular diseases [7], and liver diseases [8]. Therefore, it is essential to monitor this compound in biological samples [9]. Several analytical methods have previously been developed for the detection of MDA in biological samples, such as fluorescence [9–11], UV–visible spectrometry [12–14], surface-enhanced Raman spectra [15], mass spectrometry [16], and electron capture detection [17]. These detection techniques have been combined with some separation technologies, derivatization, or labeling to improve sensitivity and amplifying signals [18]. A derivatization based on the reaction of MDA with 2-thiobarbituric acid (TBA) is the most common method for MDA determination [19]. The drawback of this method is non-specificity [12] because TBA reacts not only with MDA but also with many other compounds present in biological samples such as sugar, aldehydes, and pyrimidine [20]. Some of these reaction products interfere with the UV–vis and fluorescence detection of TBA–MDA adduct [15]. Furthermore, high temperature of 95 °C required in the assay reaction may generate further oxidation of the matrix with obvious overestimation of the results [21, 22]. To overcome the limitation of the TBA assay, another reagents were used for malondialdehyde derivatization under mild conditions as 2,4-dinitrophenylhydrazine [19], diaminonaphthalene [13], phenylhydrazine [12]. MDA is present in biological samples at trace levels, therefore sensitive techniques as liquid or gas chromatography with UV or MS detection are used for detection of MDA after its derivatization. These techniques are expensive, time consuming and require sometimes long sample pre-treatment processes. Hence, the development of a fast, sensitive and selective method to detect MDA with simple instrument is necessary [23].

Malondialdehyde is an electroactive compound which allows its detection by electrochemical techniques. Compared to conventional methods, electrochemical methods have many advantages such as simplicity, fast response, high sensitivity, low cost and small sized commercial detectors [24]. Only a few works have been reported on the determination of MDA in biological samples by electrochemical detection [18, 23, 25, 26]. Several electrochemical techniques such as cyclic voltammetry (CV), linear sweep voltammetry (LSV), and differential pulse voltammetry (DPV) have been discussed in the literature for the detection and determination of MDA in biological samples [23, 25, 27]. The working electrodes used in these referred works involved the electrochemical sensing of malondialdehyde with glassy carbon electrodes or gold electrode, while simple miniaturized screen-printed carbon electrodes (SPCEs) are never used for this purpose.

Many works on SPCE technology have been used to develop various electrochemical sensors for detecting several compounds in the biomedical, agri-food, and environmental fields [28, 29]. SPCEs are distinguished by being low cost, small sized, and flexible and can be used as a disposable sensor. To enhance the sensitivity and the selectivity to detect different analytes, electrochemical sensors have been modified with various nanomaterials [29]. Graphene is a 2-dimensional material which possesses unique physicochemical properties such as high surface area, excellent conductivity, good electrocatalytic activity, and ease of functionalization [30]. Therefore, many electrochemical sensors using graphene, especially functionalized graphene as the sensing material, were developed for molecule detection [31]. In recent years, porphyrin-functionalized graphene–based materials have attracted a lot of attention and were used to modify working electrodes for electrochemical detection of different analytes [32]. Wu et al. [24] developed an electrochemical senor based on anion porphyrin TCPP–stabilized graphene (TCPP/CCG) for highly sensitive and selective detection of dopamine. In the same context, porphyrin-functionalized graphene–based materials are used for the electrochemical detection of dopamine by Han et al. [33] and Lv et al. [31]. In addition, Wu et al. [34] and Zhang et al. [35] used porphyrin-functionalized graphene coupled with an electrochemiluminescence technique and an electrochemical aptasensor for the ultrasensitive label-free detection of human telomerase activity and ATP, respectively.

In this work, porphyrin-functionalized magnetic graphene oxide (TCPP–MGO) was synthetized to modify the surface of screen-printed carbon electrode (SPCE). The electrochemical sensor constructed was employed for sensitive and selective determination of malondialdehyde in serum samples. Prior electrochemical detection of MDA, a derivatization procedure consisting in the reaction of MDA with 1,8-diaminonaphthalene (DAN), was carried out in order to enhance sensitivity. The electrochemical behaviors of MDA-DAN derivative on screen-printed carbon electrode (SPCE)–modified porphyrin–functionalized magnetic graphene oxide (TCPP–MGO/SPCE) were investigated by cyclic voltammetry (CV). Compared to SPCE and MGO/SPCE, CV responses of the MDA–DAN derivative obtained with TCPP–MGO were the best option, due to the *π*–*π* stacking and electrostatic attraction between the positively charged MDA–DAN derivative and negatively charged TCCP–MGO increasing MDA–DAN molecules on the surface of the electrode and thus accelerating the electron transfer. The performance of the TCPP–MGO/SPCE sensor is evaluated for the determination and detection of MDA in chicken serum samples, and the results obtained exhibited a good selectivity and high sensitivity.

## Experimental

### Instrumentation

Electrochemical detection was carried out with a CHI842D electrochemical analyzer from CH instruments (Austin, TX, USA) controlled with a computer for the control of data processing which was performed using the electrochemical analyzer software. All experiments were carried out using a screen-printed carbon electrode (SPCE) system (Metrohm Dropsens DRP-110) housed in the home-made electrochemical flow cell. Ultrapure water was obtained by a Milli-Q Plus system (Millipore, Bedford, MA, USA). An oven from Hewlett Packard 5890, Series II gas chromatograph (WA, USA), and an ultrasound bath (Selecta, Barcelona, Spain) were used. The correct MDA–DAN preparation was verified with a 6545 LC/Q-TOF (Agilent Technologies) and a Secomam Uvi Light XS 2 spectrophotometer (Alés, France). The template was purified by using a combination of a modular LC Jasco system (Easton, MD, USA) consisting of an LC ternary pump (PU-2080 Plus), a sampler (AS-2055 Plus), a column oven (CO-2065 Plus), a circular dichroism detector (JASCO CD-2095 PLUS), an Agilent LC analytical column (model Sorbax Eclipse XDB-C18, 150 mm × 4.6 mm i.d., 5 µm particle size), and a Cole-Parmer microcomputer Controlled Fraction Collector CHF122SC with a 120-position tray (Chicago, IL, USA). Data were acquired and the equipment controlled using CHROMNAV software, which was run under Microsoft Windows XP on an IBM-compatible personal computer.

XRD patterns were measured on a Philips model X’Pert MPD diffractomer using a CuKα source (*λ* = 1.5418 A), a programmable divergence slit, a graphite monochromator, and a proportional sealed xenon gas detector.

### Chemicals, reagents, solutions, and sample preparation

Malondialdehyde bis (diethyl acetal) for synthesis (MDA), 1,8-diaminonaphthalene (DAN), and meso-tetra(4-carboxyphenyl)porphin (TCPP) were obtained from Sigma-Aldrich (MO, USA) as well as iron (III) chloride hexahydrate, cobalt (II) chloride hexahydrate, tetraethyl orthosylicate (TEOS), 3-aminopropyltriethoxysilane (APTES) (99%), graphite, sodium sulfite, potassium permanganate, hydrazine solution (85%), phosphotungstic acid solution, BHT, and sodium hydroxide pellets. Hydrogen peroxide (30%) was obtained from Fisher Scientific (Madrid). Methanol (HPLC grade) was purchased from Fisher Scientific (Loughborough, Leics, UK). Ethanol, hydrochloric acid (HCl) 37%, glacial acetic acid, sulfuric acid (H_2_SO_4_) (96%), and NH_3_.H_2_O (30 wt%) were provided by Panreac Química SLU (Barcelona, Spain). Ultrapure water used in the experiments was obtained by a Milli-Q Plus system (Millipore, Bedford, MA, USA).

The standard stock solution of MDA of 1 mol L^−1^ was prepared by diluting an adequate volume of the commercial solution in MeOH and stored at 4 °C. MDA stock solution was diluted to working standard solutions with water. DAN solution was prepared at 5.8 mM in HCl 2.4 N, sonicated for 15 min, stored for 1 night at 4 °C, and then centrifuged. The supernatant obtained was stored as separate aliquots that are stable for at least 1 week at 4 °C.

Chicken serum samples were collected from healthy birds slaughtered for human consumption and stored at − 80 °C until analysis. For sample preparation, 20 µL of serum samples was gently mixed with 500 µL of 42 mM sulfuric acid in a microcentrifuge tube. Phosphotungstic acid solution 125 µL was added and was mixed by vortexing. The samples were incubated at room temperature for 5 min and then were centrifuged at 13,000 × g for 3 min. Then, the supernatant was discarded and, in a separate tube, 2 µL of BHT (100 ×) was added to 800 µL of DAN solution. Finally, the pellet was resuspended on ice with the DAN/BHT solution.

### Preparation and purification of MDA–DAN

The preparation and purification of MDA–DAN was carried out according to the previously described procedure [36]. Briefly, malondialdehyde bis(dimethyl acetal) standard solutions were hydrolyzed in water in situ to form MDA. Then, 900 μL of DAN solution prepared in HCl was added to the MDA solution. The final volume was then adjusted to 1 mL with deionized water, and the reaction mixture was kept at 37 °C for 180 min. The formation of DAN/MDA was confirmed through MS/MS and UV–vis analysis.

### Synthesis of magnetic graphene oxide porphyrin (TCPP/MGO)

The synthesis method of TCPP/MGO was previously described by Wu et al. [23]. In the first step, magnetic graphene oxide (MGO) was synthetized as described in our previously published paper [36] by incorporating magnetism [37] to GO prepared according to the Hummers method [38]. In the second step, 31.6 mg of TCPP dissolved in 0.2 mL of a 1-mol/L NaOH solution was mixed with 3 mL of a 2.7-mg mL^−1^ of MGO solution, and sufficient H_2_O to bring the total volume to 40 mL. After being ultrasonicated for 30 min, the mixture was stirred at 70 °C for 8 h. Subsequently, 6.4 μL of hydrazine solution and 128 μL of NH_3_.H_2_O were added to the mixture solution, and the resulting mixture was heated at 95 °C for 8 h under vigorous agitation. The product was washed with water and methanol several times and then dried at 50 °C.

### Preparation of modified electrode

Each of the synthetized MGO and TCPP-MGO were ultrasonically dispersed in ethanol. The concentration of the obtained suspension was 1 mg mL^−1^. Dropsens SPCEs (DRP-110), with carbon as a working electrode and a disk shape of 4 mm diameter, were used to fabricate the electrode. A volume of 2.5 µL of the dispersed TCPP-MGO or MGO was casted onto the surface of the SPCE. Prior to electrode modification, a magnet was placed below the electrode with the aim of fixing the deposited magnetic nanocomposite. Then, the modified SPCE was dried by using a hair dryer and was rinsed with pure water. Finally, the electrode was ready to use.

### Electrochemical measurements of MDA–DAN

A drop of 50 µL of MDA-DAN with an appropriate concentration was casted onto the surface of the SPCE. Cyclic voltammetry (CV) and differential pulse voltammetry (DPV) measurements were carried out. After each run, the SPCE was cleaned with ultrapure water to remove MDA–DAN for reuse and regeneration of the electrode surface. All experiments were conducted at room temperature.

## Results and discussion

### Characterization of MGO, TCPP/MGO, and MDA–DAN derivative

**Fig. 1:**
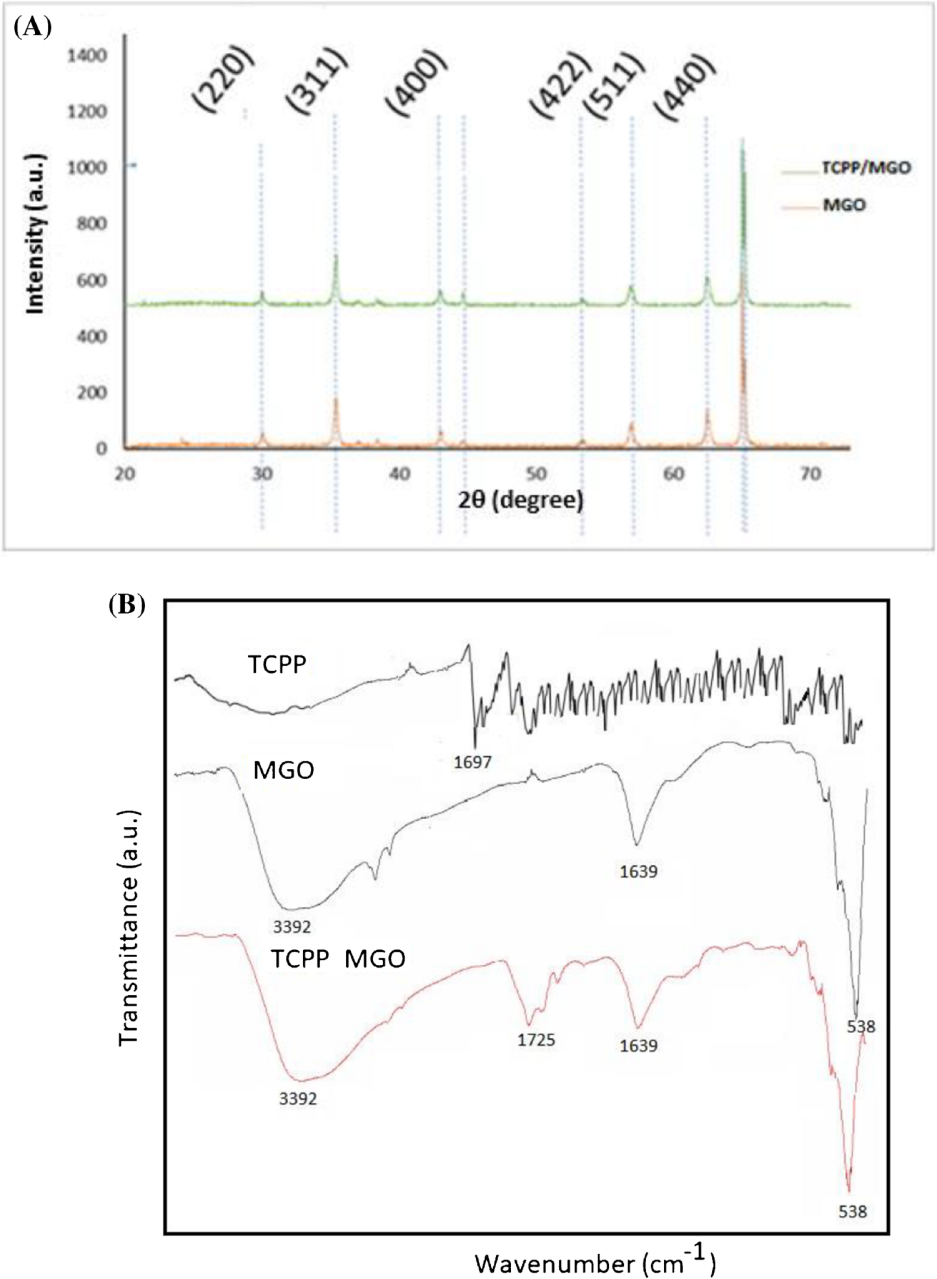
**A** XRD spectra of MGO and TCPP/MGO. Refraction planes are depicted above each peak. **B** FTIR spectra of TCPP, MGO, and TCPP-MGO composites

MGO and TCPP/MGO materials were analyzed through different techniques. X-ray diffraction (XRD) indicated that the magnetic nanoparticles remained stable throughout the whole polymerization process; as can be seen from the diffractions shown in Fig. 1A, 30.2°, 35.5°, 43.1°, 45.0°, 53.5°, 57.1°, and 62.4° are observed, all corresponding to cobalt-iron oxide nanoparticles.

The surface functional groups were investigated using FTIR analysis. Figure 1B indicates that the FTIR of the MGO before adsorption has vibrational peaks at 3392 cm^−1^ and 1639 cm^−1^ which are for the bending vibration of the –OH groups. The peak at 538 corresponds to Fe–O vibration. The peak of 1697 cm^−1^ in TCPP is ascribed to ʋ(C = O) vibration in –COOH, while in TCPP/MGO, it shifts to 1725 cm^−1^ which might be attributed to the *π*–*π* stacking and hydrophobic forces between TCPP and MGO.

The derivative resulting from the reaction of malondialdehyde (MDA) with 1,8-diaminonaphthalene (DAN) was naphtho[1,8-ef][1,4]diazepine. The mechanism of the derivatization is based on the reaction between the primary amine R-NH_2_ of diaminonaphthalene and the carbonyl group C = O of MDA under acidic conditions. This reaction is composed of four steps: Firstly, ion H^+^ resulting from ionization of hydrochloric acid (HCl) was fixed by the carbonyl group C = O of MDA. Then, a nucleophilic addition of the nitrogen of the primary amine to the electrophilic carbon of the aldehyde results in the first intermediate having a negative charge ( −) on O and a positive charge ( +) on N. The stabilization of this charged intermediate was affected by the transfer of the hydrogen atom resulting in a neutral amino alcohol. After that was the protonation of oxygen of the amino alcohol with ions H^+^ present in the acidic medium. Finally, a water molecule was eliminated and regeneration of the H^+^ catalyst occurred by the departure of the hydrogen atom to form the carbon–nitrogen double bond.

**Fig. 2:**
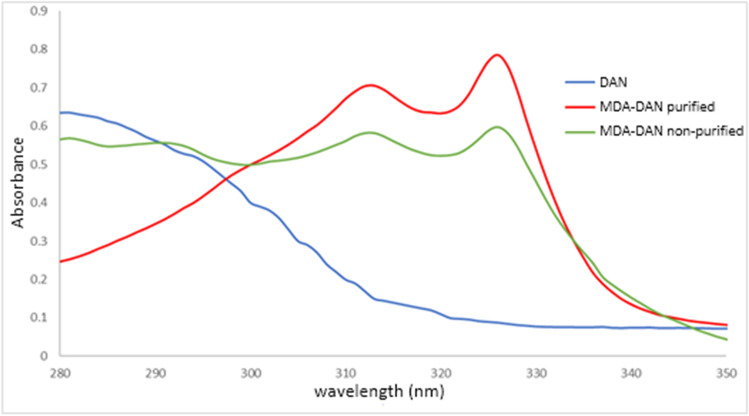
UV–vis spectra of DAN, non-purified MDA–DAN, and purified MDA–DAN solutions

The confirmation of the structure of the MDA–DAN derivative was carried out by LC/Q-TOF to obtain the MS/MS spectrum. Characteristic peaks for DAN and MDA–DAN were confirmed. MDA–DAN was chromatographically separated from the excess of DAN, allowing MDA–DAN purification after preparative chromatographic collection. UV–vis spectra confirmed the appropriated purification of MDA–DAN, free of DAN excess, according to the reported bands in the bibliography [13] and confirmed with the experimental recordings (Fig. 2).

**Fig. 3:**
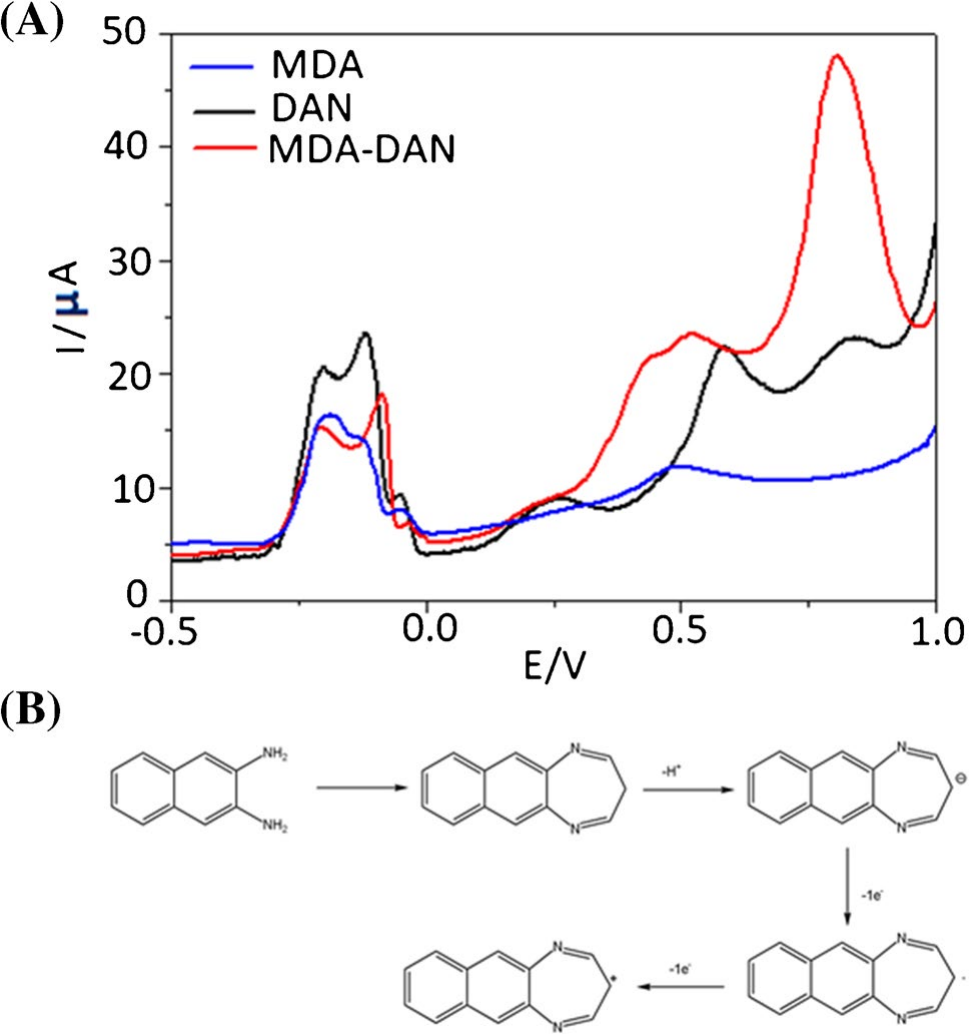
**A** DPV responses of 5.8 mM DAN, 0.1 mM MDA with 5.22 mM DAN, and 0.1 mM MDA solution using TCPP–MGO/ SPCE. **B** Oxidation mechanism of MDA–DAN

The MDA–DAN derivative was also characterized electrochemically by differential pulse voltammetry (DPV) technique. Figure 3 shows the oxidation mechanism of MDA–DAN and DPV responses of the solutions of 5.8 mM DAN, 0.1 mM MDA with 5.22 mM DAN, and 0.1 mM MDA using TCPP-MGO/SPCE. According to the results obtained (Fig. 3A), the DPV curve of 0.1 mM MDA solution shows one oxidation peak at 0.5 V with a weak current intensity of 11.9 µA. According to Toniolo et al. [39], the electrooxidation process of the aldehydes seems to proceed through similar pathways as in water electrolytes and involves, along with the dissociative chemisorption of the compounds, the formation of poisoning intermediates (such as CO, CO_2_, or carboxylic acids). The DPV curve of 5.8 mM f DAN (Fig. 3A) exhibited two oxidation peaks at 0.58 V and 0.82 V with current intensity of 22.3 and 23 µA respectively. The same oxidation peaks shown in the DPV curve of DAN were obtained in DPV curves of the MDA–DAN derivative with obvious enhancement in the current oxidation peak at 0.82 V ranging from 23 µA up to 47.8 µA (Fig. 3A). The oxidation mechanism of MDA–DAN is as represented in Fig. 3B. It is possible that in the electrochemical condition, it was generated an anion by losing a hydrogen and one electron giving the first peak (0.82 V) related to the oxidation of the anion to radical, while the second peak (0.58 V) could be due to the formation of the aromatic cation that is less energetic than the change from anion to a radical by gaining aromatization in the second oxidation.

### Electrochemical behavior of MDA–DAN on TCPP–MGO/SPCE

**Fig. 4:**
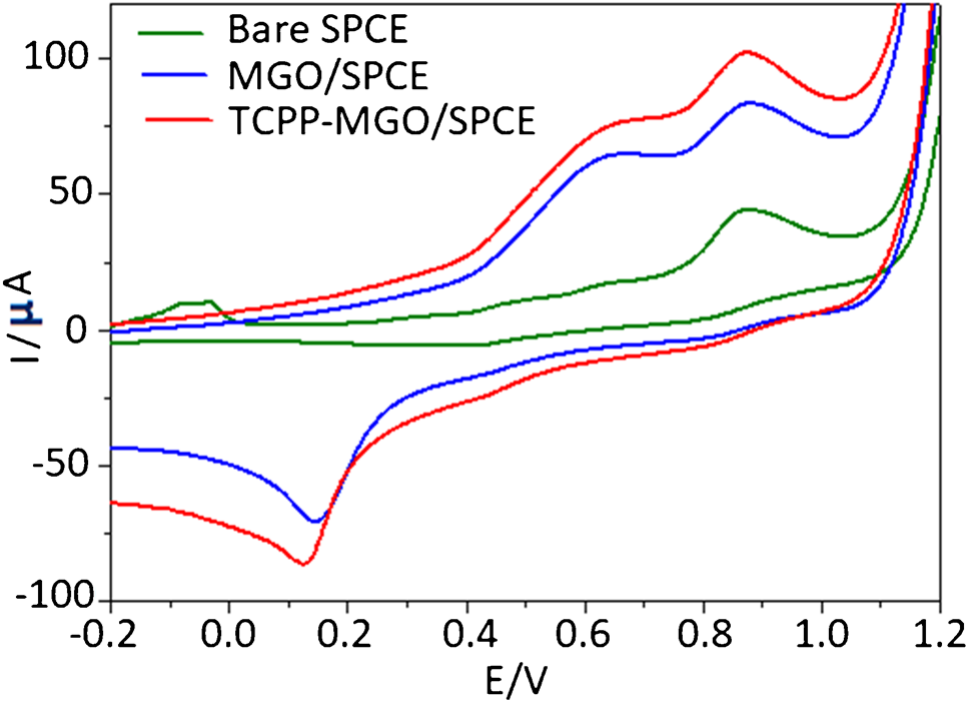
The cyclic voltammograms of different electrodes: bare SPCE, MGO/SPCE, and TCPP–MGO/SPCE at 0.1 mM MDA with 5.22 mM DAN solution and 100 mV/s scan rate

The electrochemical behavior of MDA–DAN on TCPP–MGO/SPCE has been studied under the optimum conditions using cyclic voltammetry (CV) technique. Figure 4 shows the cyclic voltammograms of 0.1 mM MDA reacting with 5.22 mM DAN at bare SPCE, MGO/SPCE, and TCPP-MGO/SPCE respectively. Table 1 summarizes the electrochemical parameters of MDA–DAN at the different electrodes. The CV responses show that only a weak oxidation peak was obtained on the bare SPCE at 0.87 V with current intensity *I*_ap_ of 44.7 µA while a pair of stronger redox peaks was observed on MGO/SPCE and TCPP-MGO/SPCE respectively. As shown in Table 1, the oxidation current of MDA–DAN on TCPP–MGO/SPCE (102 µA) was higher than that on bare SPCE (44.7 µA) and MGO/SPCE (83.5 µA). This good oxidation current should be attributed to the large electroactive surface area and fast charge transfer of GO. The derivatization of MDA with 1,8-diaminonaphthalene (DAN) also resulted in a positive aromatic electroactive molecule. TCPP, a water-soluble anion porphyrin, is strongly adsorbed on graphene through stacking, and hydrophobic interactions introduce more negatively charged –COOH groups on the graphene surface, without further destroying the conjugated system of graphene. This negatively charged surface can strongly absorb the positively charged MDA–DAN and provides a suitable environment for the MDA–DAN oxidation. Therefore, TCPP–MGO/SPCE improved the oxidation of MDA–DAN indicating that the high conductivity and high surface area of this electrode enhanced the catalytic activity and increased the effective electrode area toward MDA–DAN oxidation. Hence, TCPP-MGO/SPCE is suitable for the determination of MDA–DAN.

**Table 1:** Electrochemical parameters of MDA–DAN obtained at different electrodes

Electrodes	*E*_ap_ (V)	*I*_ap_ (µA)	*E*_cp_ (V)	*I*_cp_ (µA)	Δ*E*_p_
SPCE	0.87	44.7	–	–	–
MGO/SPCE	0.87	83.5	0.14	− 70.2	0.73
TCPP-MGO/SPCE	0.87	102	0.13	− 86.1	0.74

*E*_*ap*_ anodic peak potential, *E*_*cp*_ cathodic peak potential, *I*_*ap*_ anodic peak current, *I*_*cp*_ cathodic peak current

### Optimization of experimental parameters

The electrochemical response of the sensor toward the determination of MDA–DAN was optimized by analyzing a standard solution of 0.1 mM MDA with 5.22 mM DAN in triplicate using differential pulse voltammetry (DPV) technique. Two important parameters that affect the determination of MDA–DAN, such as TCPP–MGO amount and drop volume of MDA–DAN solution, were studied and optimized.

**Fig. 5:**
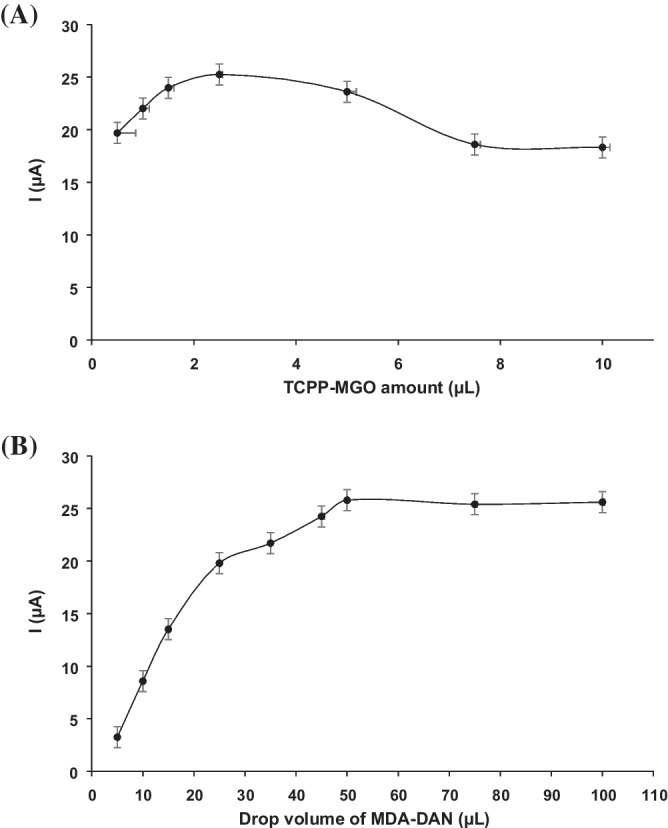
**A** Analytical signals obtained for different amounts of TCPP–MGO materials (0.5, 1.0, 2.5, 5, 7.5, 10 µL) at 0.1 mM MDA with 5.22 mM DAN solution. **B** Analytical signals obtained for different drop volumes of MDA–DAN solution (5, 10, 15, 25, 35, 45, 50, and 100 µL)

To evaluate the influence of the amount of TCPP–MGO on the SPCE surface on the determination of MDA–DAN derivative, different modification volumes were optimized from 0.5 to 10 µL. The results show that 2.5 µL gave the best result of oxidation peak current sensitivity than the others (Fig. 5A). The effect of drop volume of the MDA–DAN solution was also investigated. Different volumes were tested, 5, 10, 15, 25, 35, 45, 50, 75, and 100 µL. With the increase of the volume, the oxidation current of MDA–DAN increased from 5 to 50 and reached up the maximum at 50 µL (Fig. 5B). So, 50 µL was chosen as the optimal volume.

The stability of the TCPP–MGO/SPCE was studied by investigating the differential pulse voltammetry (DPV) responses of 0.1 mM MDA with 5.22 mM DAN at TCPP–MGO/SPCE. Five electrodes were prepared with the same procedure. The stability results showed that the remaining percentage of the initial response for MDA–DAN at TCPP–MGO/SPCE was 97%, which demonstrated a good stability of the TCPP–MGO/SPCE.

### Analytical applications

The analytical parameters for the detection of MDA using the TCPP–MGO/SPCE sensor have been investigated by measuring the DVPs of MDA–DAN with different concentrations of MDA under the optimal conditions. The calibration curve showed a linear range for standard MDA–DAN solutions of 0.01–100 µM. Table 2 summarizes the results of linear range, slope, intercept, and the regression coefficient (*R*^2^ = 0.9996) of the calibration curve of MDA–DAN. The theoretical limit of detection, defined as the concentration of analyte giving a signal equivalent to the blank signal plus three times its standard deviation, was calculated. In this work, the limit of detection (LOD) was taken to be the amount of analyte that gave a signal clearly distinguished from the instrument’s background noise. The LOD thus calculated for the proposed method was 0.007 µM. The practical limit of quantification (P-LOQ), which is defined as the minimum level at which MDA can be determined in serum samples with acceptable accuracy (> 80%) and precision (RSD < 10%), was 0.010 µM.

**Table 2:** Analytical parameters of electrochemical detection of MDA using TCPP–MGO/SPCE

Parameter	MDA
Linear range/µM	0.01–100
Calibration graph	
Intercept	0.1147 ± 0.0004
Slope	0.1604 ± 0.0013
Correlation coefficient	0.9996
Limit of detection (LOD)/ /µM	0.007
Practical limit of quantification/µM	0.010
RSD (%) (*n* = 11)^a^	6.87

^a^30 µM of MDA

The precision of the method for standard solutions (investigated after analyzing 11 series of 11 replicates) and the relative standard deviation (RSD) was calculated to be 6.87% at the 30 µM concentration of MDA.

The effect of potential interferences in the analytical signal of MDA was carried out for some common compounds in the samples to be analyzed, such as Ca^2+^, Mg^2+^, PO_4_^3−^, 4 − hydroxynonenal, and glutaraldehyde. This study was carried out with a solution of MDA 5 µM. A compound was considered to interfere if a variation of more than 5% was observed in the analytical signal. All the results are shown in Table 3. It can be observed that no interferences were observed at the tested interferent/analyte ratios.

**Table 3:** Effect of foreign species

Foreign species	Tolerated interferent/analyte (w/w) ratio^a^
Ca^2+^, PO_4_^3−^, glutaraldehyde	> 250^b^
Mg^2+^, 4 − hydroxynonenal	> 150^b^

^a^For MDA concentration of 5 µM

^b^Maximum ratio tested

The TCPP–MGO/SPCE sensor was applied for determination of malondialdehyde in three different chicken serum samples. Firstly, three concentrations of MDA were applied by standard addition method of each serum sample, to obtain the intercept, and through this, the concentration of MDA in each chicken serum sample, in which no MDA was detected. Plasma samples were spiked with MDA–DAN at different concentration levels. The analytical applications were evaluated in triplicate by differential pulse voltammetry under the optimized conditions. The concentration of MDA in the spiked serum samples was calculated from the calibration equation. The results obtained are summarized in Table 4. It is shown that recoveries ranged from 94 to 106% (errors between 3.3 and 6.5%).

**Table 4:** Recovery results for the determination of MDA in spiked chicken serum samples at different levels using the TCPP-MGO/SPCE sensor

Chicken serum samples	Spiked concentration (µM)	Found concentration (µM)	Recovery (%)
Sample 1	0.020	0.020 ± 0.001	100
	0.500	0.531 ± 0.005	106
	5.000	5.305 ± 0.020	106
	10.000	9.412 ± 0.023	94
Sample 2	0.020	0.019 ± 0.001	95
	0.500	0.474 ± 0.003	95
	5.000	5.351 ± 0.012	107
	10.000	9.520 ± 0.041	95
Sample 3	0.020	0.021 ± 0.001	105
	0.500	0.474 ± 0.023	95
	5.000	4.842 ± 0.087	97
	10.000	9.583 ± 0.102	96

In Table 5, a comparative study of some previous electrochemical alternative methods reported in the bibliography for the determination of MDA can be observed [18, 25, 36, 40]. In general, the estimated LOQ and RSD values were significantly lower than or in the same order as those of the other reported methods. Moreover, this method used one of the most extended linear ranges, presenting one of the best analytical features.

**Table 5:** Comparative study of some electrochemical methods for MDA determination

Electrode/modifier	LOQ (µM)	Precision (%)	Linear range (µM)	Reference
GCE^a^/RF-PT-AgNPs^b^	590	11	620–890	39
Au/ PT^c^	0.02	15	0.02–3	25
GCE^a^/MWCNTs	0.1	9	0.02–40	18
SPCE/MGO@MIPy^d^	0.01	4	0.01–100	36
SPCE/TCPP–MGO	0.01	7	0.01–100	This work

^a^Glass-carbon electrode

^b^Self-assembled riboflavin–taurine coupled with silver nanoparticles

^c^Polytaurine

^d^Magnetic graphene molecularly imprinted polypyrrole polymer

## Conclusions

The developed TCPP–MGO/SPCE sensor exhibited high sensitivity and a wide linear range for the determination of MDA–DAN derivative with a low practical limit of quantification (P-LOQ), 0.01 µM. *π*–*π* stacking and electrostatic attraction between the positively charged MDA–DAN derivative and negatively charged porphyrin-functionalized magnetic graphene oxide increased the interactions of the MDA-DAN derivative to the surface electrode and accelerated the electron transfer. The performance of the TCPP-MGO/SPCE sensor demonstrated its usefulness for the detection and determination of MDA in serum samples, with good selectivity in spite of the complex matrix. With this work, new possibilities are shown for the design of electrochemical sensors based on functionalized GQDs depending on the specific chemistry of various target analytes to also find an effective resolution of the mixtures in complex samples with an improved sensitivity.
